# A first-in-human study of the novel HIV-fusion inhibitor C34-PEG_4_-Chol

**DOI:** 10.1038/s41598-017-09230-0

**Published:** 2017-08-25

**Authors:** Killian Quinn, Cinzia Traboni, Sujan Dily Penchala, Georgios Bouliotis, Nicki Doyle, Vincenzo Libri, Saye Khoo, Deborah Ashby, Jonathan Weber, Alfredo Nicosia, Riccardo Cortese, Antonello Pessi, Alan Winston

**Affiliations:** 1 Department of Medicine, Imperial College London, London, W2 1NY UK; 2 JV Bio, Via Gaetano Salvatore 486, 80145 Napoli, Italy; 3 Department of Pharmacology, University of Liverpool, Liverpool, L69 3BX UK; 4 School of Public Health, Imperial College London, London, UK; 5 Institute of Neurology, University College London, London, WC1N 3BG UK; 6 CEINGE, Via Gaetano Salvatore 486, 80145 Napoli, Italy; 7 PeptiPharma, Viale Città D’Europa 679, 00144 Roma, Italy

## Abstract

Long-acting injectable antiretroviral (LA-ARV) drugs with low toxicity profiles and propensity for drug-drug interactions are a goal for future ARV regimens. C34-PEG_4_-Chol is a novel cholesterol tagged LA HIV-fusion-inhibitor (FI). We assessed pre-clinical toxicology and first-in-human administration of C34-PEG_4_-Chol. Pre-clinical toxicology was conducted in 2 species. HIV-positive men were randomised to a single subcutaneous dose of C34-PEG_4_-Chol at incrementing doses or placebo. Detailed clinical (including injection site reaction (ISR) grading), plasma pharmacokinetic (time-to-minimum-effective-concentration (MEC, 25 ng/mL) and pharmacodynamic (plasma HIV RNA) parameters were assessed. In both mice and dogs, no-observed-adverse effect level (NOAEL) was observed at a 12 mg/kg/dose after two weeks. Of 5 men enrolled, 3 received active drug (10 mg, 10 mg and 20 mg). In 2 individuals grade 3 ISR occurred and the study was halted. Both ISR emerged within 12 hours of active drug dosing. No systemic toxicities were observed. The time-to-MEC was >72 and >96 hours after 10 and 20 mg dose, respectively, and mean change in HIV RNA was −0.9 log10 copies/mL. These human pharmacodynamic and pharmacokinetic data, although limited to 3 subjects, of C34-PEG-4-Chol suggest continuing evaluation of this agent as a LA-ARV. However, alternative administration routes must be explored.

## Introduction

Mounting evidence from both high and low income countries suggests a near normal life expectancy for people living with HIV (PLWH) who start antiretroviral therapy with CD4+ lymphocyte cell counts above 350 cells/µL^1^. Consequent to the success of antiretroviral therapy and the longevity expected for PLWH, over recent decades there has been a gradual upward shift in the median age of PLWH. Antiretroviral therapy management becomes increasingly challenging in older individuals due to the presence of non-infectious co-morbidities^2^, which are reported to occur at a higher incidence in PLWH compared to matched control populations, the presence of poly-pharmacy and drug-drug interactions. As such, there is an urgent unmet need for new antiretroviral agents and combinations for older PLWH which includes drugs with a low propensity for drug-drug interactions and which lack end-organ toxicities^3^.

The HIV-fusion inhibitors (FIs) are peptides derived from the heptad repeat (HR)−2 region of the viral fusion protein gp41. Their mechanism of action is to prevent the formation of a critical intermediate along the virus-cell fusion pathway responsible for enacting cell-virus fusion. To date, only one FI, enfuvirtide has been approved for use^4, 5^. Clinical use of enfuvirtide has been limited by the lack of oral bioavailability and short half-life, thus necessitating twice daily subcutaneous injections, and high rates of painful injection site reactions (ISRs). Notwithstanding, enfuvirtide and other investigational FIs display low systemic toxicity and a general lack of drug-drug interactions, making them promising for further investigation as antiretroviral agents^4–6^. Modification of a number of FI compounds has prolonged their plasma half-lives in animal models and fueled a renewed interest in their development as potentially long-acting antiretroviral drugs suitable for use in older PLWH with comorbidities and receiving concomitant medications^7–9^.

Extensive evidence supports that HIV viral entry occurs within cholesterol and sphingolipid enriched cell membrane domains known as ‘lipid rafts’^10, 11^. Notably, CD4+ receptors, the primary receptors on HIV target cells, lie within lipid rafts on the target membrane. In addition, gp41 associates with caveolin-1, the structural protein component of a subset of lipid rafts known as caveolae. C34 is a lead compound corresponding to amino acid residues 117–150 of the HR-2 region of gp41. By conjugating to C34 a cholesterol group with a 4-unit polyethylene glycol (PEG_4_) spacer, the compound is concentrated in lipid rafts of cell membranes. Compared with underivatised C34, C34-PEG_4_-Chol shows dramatically increased antiviral potency on a panel of primary isolates, with 90% maximal inhibitory concentration (IC_90_) values 15- to 300-fold lower than enfuvirtide. With an IC_90_ between 15–460 pM (0.08–2.5 ng/mL) depending on the viral strain, C34-PEG_4_-Chol is the most potent HIV fusion inhibitor to date^12^. Moreover, the cholesterol moiety drives binding to serum proteins, an effective way to improve peptide pharmacokinetics^13^ and accordingly, the circulatory half-life of C34 in rodents is extended by 10-fold when conjugated with cholesterol^12^. Allometric scaling from animal studies suggests that once-weekly subcutaneous administration in humans may be achievable with a dose range of 10–80 mg.

We sought to establish the safety, pharmacokinetic profile and pharmacodynamic effects of C34-PEG_4_-Chol as a potential long acting FI in PLWH. Here we report the pre-clinical development of C34-PEG_4_-Chol and a first-in-human study.

## Methods

### Pre-clinical methods

#### Peptide synthesis and formulation

**Figure 1:**
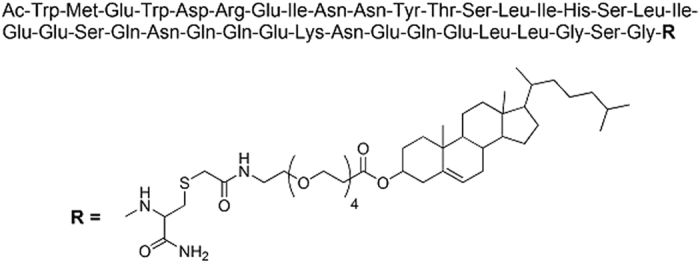
Structure of C34-PEG_4_-Chol.

The Structure of C34-PEG_4_-Chol is shown in Fig. 1. The peptide was synthesized under good manufacturing practice (GMP) conditions by American Peptide Company (Vista, CA, USA), through solid phase Fmoc/tBu chemistry, followed by chemoselective thioether conjugation between the cysteine-containing C34 precursor (Ac-WMEWDREINNYTSLIHSLIEESQNQQEKNEQELLGSGC) and a bromoacetyl cholesterol derivative, as previously described for the non-GMP synthesis^14^. The peptide was purified by reverse-phase high-pressure liquid chromatography (HPLC) and characterized by mass spectrometry, amino acid analysis, and analytical HPLC. The peptide was formulated by Symbiosis (Stirling, UK). Briefly, the purified peptide was dissolved in 0.1 M Sodium Phosphate Buffer, pH 7.4, containing 15% w/v of 2-Hydroxypropyl-β-cyclodextrin, sterile-filtered and lyophilized in individual vials. The lyophilised powder was stored at −20 °C, and reconstituted in water for injection immediately before use.

#### Pre-clinical toxicology analysis

Pre-clinical toxicology studies were conducted in 2 species (rodent/mice and non-rodent/dogs). Multiple high doses of C34-PEG_4_-Chol, up to 12 mg/kg or 10 x the maximum proposed human dose, were administered daily in mice and twice weekly (the intended frequency in humans) in dogs for 14 days, with a 7-day recovery period for both species. Both species were observed for clinical signs including ISRs, clinical pathological parameters (full blood picture, coagulation, biochemistry) and dogs additionally had electrocardiograms (ECGs) performed pre-dosing and 5 h post each dose. Toxicology results were analysed independently by the Medicines and Healthcare Regulatory Authority (MHRA), UK before a decision was made to proceed with trial in humans.

#### *In vitro* HIV-resistance

The HIV laboratory strain, BaL, was grown in a T-cell line (PM-1) starting at the IC_90_ of the C34-PEG_4_-Chol. Viral growth was monitored by a performance enhanced reverse transcriptase (PERT) assay. With each passage, drug concentration was doubled. Sequence analysis of gp41 was undertaken to identify evolution of mutations. Site-directed mutagenesis was used to create viral clones to confirm the effect of emergent mutations on drug susceptibility.

### Clinical Methods

#### Human study design

In a phase 1 (first-in-man) double-blind, randomised, placebo-controlled trial, an initial dose escalation and safety phase was planned exploring a single administration of C34-PEG_4_-Chol dose range 10–80 mg. Subsequent to this initial phase a multiple dosing study was planned. Due to the clinical studies being stopped secondary to observed toxicities, in this manuscript we report the findings from the single dose experiments. Methodological details including sample size calculations on the planned multiple dosing phase have been reported^15^.

#### Randomisation and masking

Four cohorts of participants (n = 4 per cohort; 2 participants to receive C34-PEG_4_-Chol and two to receive placebo) across four doses (10 mg, 20 mg, 40 mg, 80 mg) were planned. Each participant received a single subcutaneous dose of either C34-PEG_4_-Chol or placebo (saline). Dosing was commenced at the lowest dose of 10 mg in a volume of 1 mL. Randomisation was undertaken using the InForm™ Integrated Trial Management system and was on a 1:1 basis, with no predefined stratifications. All clinical study staff were blinded. Clinical research pharmacists who were not part of the clinical research team, were not blinded in order to reconstitute study products. These members of the research pharmacy team had no involvement in the clinical care of participants during study procedures.

Intensive safety and pharmacokinetic assessments were carried out in an in-patient hospital setting for the first 24 hours post-dosing and all participants were followed up as out-patients for 84 days. The study was approved by the National UK Research Ethics Committee (references number 14/LO/2078, EudraCT number 2014-002671-28) and the MHRA, UK. All subjects provided written informed consent prior to any study procedures being undertaken and all study procedures were conducted in keeping with Good Clinical Practice Research Guidelines (GCP).

#### Study population

This study was conducted at the Clinical Trials Centre (St. Mary’s Hospital, London) and the Imperial Clinical Research Facility (Hammersmith Hospital, London) both within Imperial College Healthcare NHS Trust, London, UK. Men aged 18 to 60 years with documented HIV-1 infection for ≥6 months were eligible. Further inclusion criteria included plasma HIV RNA ≥10,000 copies/mL, CD4+ lymphocyte count ≥400 cells/µL and negative urine screening sample for recreational drugs of abuse (Multi-Drug One Step Screen Test Panel, InstAlert™). Exclusion criteria included receipt of antiretroviral therapy ≤6 months from screening and those with previous enfuvirtide exposure or significant antiretroviral drug resistance.

#### Safety assessments

The single dose of C34-PEG_4_-Chol was administered subcutaneously lateral to the umbilicus. All participants were observed in a dedicated inpatient clinical research facility for 24 hours after dosing and thereafter, subjects were reviewed at days 2, 3, 4, 7, 10, 14, 21, 28 and 84. A modified grading system of ISRs was developed (Supplementary Information *section 1*) with reference to grading conducted in pivotal phase 3 studies of enfuvirtide^5^. At all study time-points, participants were reviewed for ISRs, a symptom-directed physical examination was performed and blood samples were collected for full blood picture, electrolytes, liver enzymes, amylase and glucose. An electrocardiogram was performed pre-dose and 4 and 12 hours after dosing. A decision to proceed with dose escalation was made only after all four participants in each dose cohort had been observed for a minimum of 7 days for safety assessments. All safety data were reviewed by an independent data monitoring committee (IDMC).

#### Pharmacokinetic evaluation

Intensive pharmacokinetic sampling was performed over the first 24 hours subsequent to dosing with samples collected pre-dose, 0.5 h, 1 h, 2 h, 4 h, 6 h, 8 h, 12 h, 24 h. Thereafter pharmacokinetic sampling was undertaken on days 2, 3, 4, 7, 10, 14, 21 and 28. Plasma samples (100 µL) were extracted in 300 µL of acetonitrile containing 1% formic acid; vortexed and centrifuged at 4000 rpm for 10 minutes. The supernatant was transferred into autosampler vials, and 25 μL injected onto the LC-MS/MS. Quantitative analysis of C34-PEG_4_-Chol was performed by LC-MS/MS using a Thermo Quantum Access triple quadrupole mass spectrometer, interfaced with a heated electrospray ionisation (H-ESI) source (Thermo Fisher Scientific, Hemel Hempstead, UK). The HPLC system included a variable loop Accela autosampler and an Accela pump (ThermoScientific, Hemel Hempstead, UK). Chromatographic separation was achieved using a reverse-phase XBridge Peptide BEH C18 column (3.5 µm; 50 mm × 2.1 mm) (Waters Corporation, Milford, US) interfaced with a 2 µm C18 Quest column saver (Thermo Scientific, Hemel Hempstead, UK). The gradient mobile phase, consisting of a solvent A (0.1% formic acid in Water) and solvent B (0.1% formic acid in acetonitrile), was delivered at a flow rate of 5000 μL/min. Detection of C34-PEG_4_-Chol was performed in the pseudo Selected Reaction Monitoring mode, monitoring a triple charged molecular ion [M + 3 H]3 + at m/z 1756.8. The internal standard (quinoxaline), was monitored at m/z 313.1 → 246.1. Peak area ratios of compound to internal standard were utilized to construct a calibration curve with a weighting of 1/x2. Data acquisition and processing was performed using LCQuan software (Version 2.7, Thermo Scientific, Hemel Hempstead, UK). The assay calibration range was between 62.5–10,000 ng/mL; inter and intra-assay accuracy and precision at the assay limit of quantification (LLQ) fell within the designated ±20% of the nominal value, and were within ±15% for all QC levels.

Pharmacokinetic parameters were derived via non-compartmental modeling. The area-under-the-concentration-time-curve (AUC) was calculated following a single dose, assuming linear decrease over time, by linear trapezoidal method. The maximum concentration (C_max_) observed at each dose and the time-to-C_max_ (T_max_) were calculated. The time to reach minimum effective concentration was assessed in order to determine the frequency of future dosing regimens. The minimum effective concentration (MEC) was defined as 10 times the IC_90_ on the hardest-to-neutralize viral strain, i.e. 25 ng/mL. Given our assay cut-off was 62.5 ng/mL, our assay cut off was used as a surrogate for the IC_90_.

#### Efficacy evaluation

Pharmacodynamic activity was assessed via changes in plasma HIV RNA (Roche Amplicor™ assay with a lower limit of quantification of 20 copies/mL). Samples for HIV RNA quantification were collected at screening, pre-dosing and days 1, 4, 7, 14 and 28. Changes in plasma HIV RNA were assessed as changes from baseline in log_10_ plasma HIV RNA load and maximum change from baseline in log_10_ plasma HIV RNA. HIV RNA sequencing was undertaken on days 14 and 90 and compared to any resistance mutations observed to evolve in the *in vitro* experiments and mutations reported to evolve with enfuvirtide^16^.

#### Stopping rules

Study stopping criteria were a serious adverse event occurring in one participant, grade 3 or 4 clinical or laboratory adverse events occurring on at least two participants or moderate intensity events occurring in three or more participants.

## Results

### Pre-clinical results

#### Pre-clinical toxicity data

Repeat-dose toxicity studies were conducted in mice (n = 20 at 6 mg/kg/day, n = 20 at 12 mg/kg/day) and dogs (n = 6 at 6 mg/kg/day, n = 6 at 12 mg/kg/day). The subcutaneous route of administration was selected for both species. The highest dose of 12 mg/kg/day was based on the maximal achievable dose with the formulation, and corresponded to 10x the maximum proposed human dose (80 mg, 1.15 mg/kg). No premature decedents occurred in either species. Following observation, animals were euthanised.

The peptide was well tolerated in mice up to 12 mg/kg/day, with no adverse clinical signs, body weight changes, effects on food consumption, ophthalmic changes or any other clinical pathology changes; no findings were noted in tissues examined histopathologically. The no-observed-adverse-effect-level (NOAEL) was therefore established at 12 mg/kg/day.

Similarly in dogs C34-PEG_4_-Chol was well tolerated, with no adverse clinical signs or toxicologically significant findings, with the exception of some subcutaneous inflammation at the injection sites in all groups, including controls, with greater severity in animals dosed with the peptide. The inflammation was reversible at the end of the recovery period. There was no evidence of systemic toxicity and no changes were observed in cardiology parameters, respiration or in motor activity at any dose level at any time during the study. See Supplementary Information (*section 2*) for further details.

Based on this, NOAEL in dogs was also considered 12 mg/kg/dose.

#### *In vitro* HIV-resistance

Ten viral passages were conducted (up to 67.4 ng/mL) whereupon viral growth was completely inhibited. A single point mutation arose within the heptad repeat (HR)−1 domain of gp41 (A71T) and two compensatory mutations within the HR-2 domain (E136G, E148K). These mutations differ from enfuvirtide where mutations emerge rapidly at positions 36–43 of gp41 with occasional compensatory mutations at 136–138^17^. Viruses containing A71T, E136G and E148K remained replication competent. Clonal virus containing A71T mutation allowed cell entry in the presence of 5.3 ng/mL of C34-PEG_4_-Chol.

### Clinical results

#### Baseline characteristics

Between March and September 2015, 13 subjects underwent screening and five were enrolled. Reasons for screen failures included raised alanine-transaminase (n = 3), body mass index above 28 (n = 1), positive urine drug of abuse screen (n = 1), CD4 count <400 cells/µL (n = 2) and HIV RNA <10,000 copies/mL (n = 1). All five participants completed 84 days of follow up. These patients’ baseline characteristics are described in Table 1 and of these 5 participants 2 received placebo, 2 received 10 mg C34-PEG_4_-Chol and 1 received 20 mg C34-PEG_4_-Chol.

**Table 1:** Baseline characteristics.

Parameter	Participant number				
	1	2	3	4	5
Age (years)	60	28	33	19	32
Ethnicity	White	White	Mixed Race	Black African	White
CD4 count (cells/µL)	486	496	478	407	1085
Plasma HIV RNA (copies/mL)	64,763	20,556	26,518	12,168	44,283
Height (cm)	168	175	172	176	170
Weight (kg)	73	66	59	63	74
BMI	26	21	20	20	26
ART history	Naive	Naive	Naïve	Experienced	Naive
Dose allocation (mg)	10	10	10	10	20
Treatment allocation	A	P	A	P	A
A = active					
P = placebo					

#### Safety and tolerability

Two grade 3 ISRs occurred in subjects administered active product (one patient receiving 10 mg and one receiving 20 mg C34-PEG_4_-Chol). The injection site reactions were delayed reactions occurring several hours after drug administration (within 12 hours) with the intensity of the reaction peaking several hours to days after the administration of the product. The grade 3 criteria was met due to the size of the ISR in the participant administered 10 mg C34-PEG_4_-Chol (maximum size 4 cm by 15 cm) and degree of tenderness and pain (interfering with usual daily activities) in the participant administered 20 mg C34-PEG_4_-Chol. No systemic clinical toxicities or laboratory defined toxicities were observed. No ISRs occurred in subjects administered placebo.

#### Pharmacokinetic measurements

**Figure 2:**
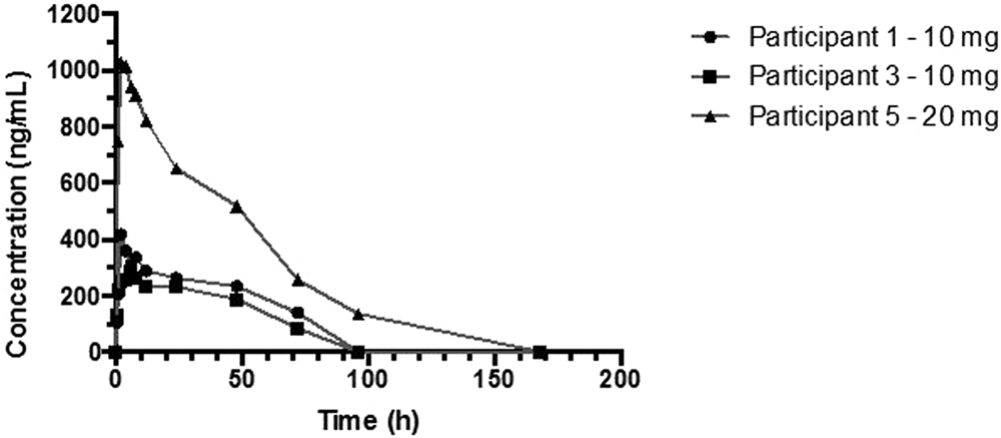
C34-PEG_4_-Chol pharmacokinetic time curves.

Plasma concentration versus time curves following administration of C34-PEG_4_-Chol to the three participants are shown in Fig. 2 and pharmacokinetic parameters listed in Table 2. The half-life ranged from 46–54 hours. The mean plasma AUC following administration of 10 mg, 10 mg and 20 mg to the three participants respectively were 247.3, 278.1 and 506.0 ng/h/mL, respectively. Plasma concentrations remained above the minimum effective concentration for 72 hours and 96 hours in subject administered 10 mg and 20 mg, respectively.

**Table 2:** Pharmacokinetic parameters.

Pharmacokinetic parameter	Participant number		
	1	3	5
Dose (mg)	10	10	20
Maximum observed concentration, (ng/mL)	417.5	278.1	1026.7
Time to maximum observed concentration (hours)	2	6	2
Area under plasma-concentration-time-curve, (ng/h/mL)	247.3	199.5	506
Half-life (hours)	48	54	46
Volume of distribution (L)	24	39.5	19.5

#### Measurements of antiretroviral activity

**Figure 3:**
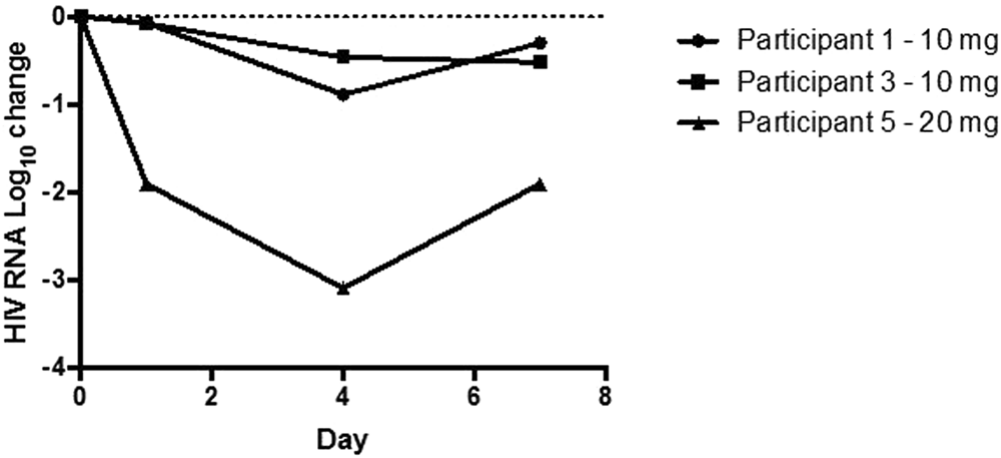
Plasma HIV RNA change from baseline after exposure to a single dose of C34-PEG_4_-Chol.

There was a substantial decline in plasma HIV RNA from day 0 to day 7 of the study in all three subjects with the maximum decline in HIV RNA observed at day 4 in all (Fig. 3). For the two participants who received 10 mg, a −0.89 Log_10_ and a −0.46 Log_10_ decline was observed from baseline. Although a highly significant drop in HIV RNA of −3.10 Log_10_ was observed on day 4 after administration of 20 mg to participant 5, review of pre-study HIV RNA levels suggested a lower set-point HIV RNA load was normal for this participant thus mitigating the magnitude of the observed antiviral effect in this participant. No HIV resistance mutations emerged over 90 days.

## Discussion

We report on pre-clinical and the first clinical study of C34-PEG_4_-Chol. Systemically, the peptide was well tolerated with no alteration in safety parameters observed. However, we observed ISRs meeting grade 3 adverse event criteria in 2 out of 3 individuals administered C34-PEG_4_-Chol via the subcutaneous route. In the pre-clinical studies, ISRs had not been observed in mice and were mild and reversible in dogs. In this species ISRs, albeit milder, were also observed in the control group, suggesting that the chosen formulation might have contributed to the side-effect. Although high rates of ISRs are observed with subcutaneous administration of enfuvirtide, and therefore not an absolute impediment for clinical use, in the present scenario of HIV therapeutics we do not consider this route of administration suitable for further clinical development. Rather, intramuscular administration might represent a viable option, in light of recent data showing that rates of ISR are less marked when long-acting rilprivirine, a non-nucleoside reverse transcriptase inhibitor is administered via intramuscular administration compared to subcutaneous^18^. Identification of both an administration route and a different formulation for C34-PEG_4_-chol that minimises ISRs would be attractive, since the preliminary pharmacokinetic and pharmacodynamic profile of C34-PEG_4_-Chol we have observed in HIV-positive men are supportive of continuing evaluation of this drug.

At a dose of 10 mg of C34-PEG_4_-Chol, we observed in two subjects a plasma concentration above the LLOQ of 62 ng/mL at 72 hours (Fig. 2). This concentration is still 25–770 fold the IC_90_ observed *in vitro* on several viral strains and notably, it coincides with the threshold (67 ng/mL) which prevents growth of resistant virus *in vitro*. In the only subject at the 20 mg dose, LLOQ was reached after 96 h. We have observed linear dose-exposure in animals, and limited to the two doses here, also in humans (Table 2). Extrapolation to higher doses, up to the intended maximum of 80 mg, strongly suggests the possibility of once-weekly use, although such extrapolations are based on data from only 3 subjects. For comparison, enfuvirtide requires administration of a  90 mg dose twice-daily. The preliminary pharmacodynamic data are also encouraging, with ≥0.5 Log_10_ reduction in plasma HIV RNA observed in all three participants after a single dose at the lower end (10–20 mg) of the intended dose range (10–80 mg) (Fig. 3). Notably, rebound of viral load was measured only after 96 hours, which is consistent with a plasma concentration of C34-PEG_4_-Chol above the IC_90_ for longer than our assay could measure. Our pharmacokinetic and pharmacodynamic observations are limited to three subjects, and therefore lack statistical power to infer such observations would be observed with larger numbers of subjects.

The first human data with a cholesterol-conjugated FI reported here augur well for the whole class. Cholesterol-conjugated FIs have been reported for several viruses for which there are limited or no therapies, which renders the issue of ISRs less relevant than for HIV therapeutics. These include retroviruses, paramyxoviruses, coronaviruses, orthomyxoviruses, henipaviruses, and filoviruses: examples include Parainfluenza virus^19, 20^, Influenza Virus^21^, Nipah Virus^19, 20, 22^, Hendra Virus^19^, SV5 (HPIV5)^19^, Measles Virus^23^, Ebola Virus^24^, Newcastle Disease Virus (NDV)^25^, and Infectious Bronchitis Virus^25^. In all cases, cholesterol conjugation improved the FI potency 50–100 fold^26^. For some of these viruses, efficacy was demonstrated *in vivo* ^22, 23, 25^ and notably, the peptide was detectable in the brain 24 hours after administration^22, 23, 25^, indicating that cholesterol conjugation may enable penetration of the blood-brain barrier, a difficult feat for drugs in general, and for biologics in particular^27^.

The observed improvement of pharmacokinetics through cholesterol conjugation is due to increased binding to serum proteins^13, 28–30^. This is a general, sequence-independent mechanism, and accordingly the preclinical pharmacokinetic data for C34-PEG_4_-Chol in mice^14^ are comparable to those of a cholesterol-conjugated Nipah virus FI in golden hamsters^22^ and a cholesterol-conjugated NDV FI in chickens^25^. Therefore the optimal pharmacokinetic profile observed in humans for C34-PEG_4_-Chol suggests that similar data are likely to be observed for other cholesterol-conjugated FIs.

It has been suggested that the improved efficacy and improved pharmacokinetics provided by cholesterol conjugation offer the basis for the rapid development of cholesterol-conjugated therapeutics for known and emerging viral diseases^26^. In particular for the threat of emerging viral diseases, it should be noted that FIs can be developed rapidly because their design only requires knowledge of the viral genome, which is available before or rapidly acquired at the time of an epidemic. Moreover, since for each viral family the fusion proteins, from which the FIs are derived, share the same mechanism of action and display considerable sequence conservation, a cholesterol-conjugated FI for a “sentinel virus” would represent the template for all the other viruses within the same family.

Although our study included small numbers of participants, the pharmacokinetic and pharmacodynamic signals were have observed in humans for C34-PEG_4_-Chol now offer a critical validation for this strategy.

## Electronic supplementary material

supplementary information

## References

[1] Samji H (2013). Closing the gap: increases in life expectancy among treated HIV-positive individuals in the United States and Canada. PLoS One.

[2] Schouten J (2014). Cross-sectional comparison of the prevalence of age-associated comorbidities and their risk factors between HIV-infected and uninfected individuals: the AGEhIV cohort study. Clin Infect Dis.

[3] Winston A, Underwood J (2015). Emerging concepts on the use of antiretroviral therapy in older adults living with HIV infection. Curr Opin Infect Dis.

[4] Lalezari JP (2003). Enfuvirtide, an HIV-1 fusion inhibitor, for drug-resistant HIV infection in North and South America. N Engl J Med.

[5] Lazzarin A (2003). Efficacy of enfuvirtide in patients infected with drug-resistant HIV-1 in Europe and Australia. N Engl J Med.

[6] Lalezari JP (2005). T-1249 retains potent antiretroviral activity in patients who had experienced virological failure while on an enfuvirtide-containing treatment regimen. J Infect Dis.

[7] Chong H (2012). Biophysical property and broad anti-HIV activity of albuvirtide, a 3-maleimimidopropionic acid-modified peptide fusion inhibitor. PLoS One.

[8] He Y (2008). Design and evaluation of sifuvirtide, a novel HIV-1 fusion inhibitor. The Journal of biological chemistry.

[9] Meng Q (2014). Pharmacokinetics of sifuvirtide in treatment-naive and treatment-experienced HIV-infected patients. Journal of pharmaceutical sciences.

[10] Aloia RC, Jensen FC, Curtain CC, Mobley PW, Gordon LM (1988). Lipid composition and fluidity of the human immunodeficiency virus. Proc Natl Acad Sci USA.

[11] Aloia RC, Tian H, Jensen FC (1993). Lipid composition and fluidity of the human immunodeficiency virus envelope and host cell plasma membranes. Proc Natl Acad Sci USA.

[12] Ingallinella P (2009). Addition of a cholesterol group to an HIV-1 peptide fusion inhibitor dramatically increases its antiviral potency. Proc Natl Acad Sci USA.

[13] Zhang, L. & Bulaj, G. Converting peptides into drug leads by lipidation. *Curr. Med. Chem*. **19**, 1602–1618, doi:CDT-EPUB-20120229-002 (2012). 10.2174/092986712799945003 22376031

[14] Ingallinella P (2009). Addition of a cholesterol group to an HIV-1 Peptide Fusion Inhibitor dramatically increases its antiviral potency. Proc. Natl. Acad. Sci. USA.

[15] Mason AJ (2017). Developing a Bayesian adaptive design for a phase I clinical trial: a case study for a novel HIV treatment. Stat Med.

[16] Shafer RW, Schapiro JM (2008). HIV-1 drug resistance mutations: an updated framework for the second decade of HAART. AIDS Rev.

[17] Rimsky LT, Shugars DC, Matthews TJ (1998). Determinants of human immunodeficiency virus type 1 resistance to gp41-derived inhibitory peptides. J Virol.

[18] Williams PE, Crauwels HM, Basstanie ED (2015). Formulation and pharmacology of long-acting rilpivirine. Curr Opin HIV AIDS.

[19] Porotto M (2010). Viral Entry Inhibitors Targeted to the Membrane Site of Action. J. Virol..

[20] Pessi A (2012). A General Strategy to Endow Natural Fusion-protein-Derived Peptides with Potent Antiviral Activity. PLoS One.

[21] Lee KK (2011). Capturing a Fusion Intermediate of Influenza Hemagglutinin with a Cholesterol-conjugated Peptide, a New Antiviral Strategy for Influenza Virus. J. Biol. Chem..

[22] Porotto M (2010). Inhibition of Nipah Virus Infection *In Vivo*: Targeting an Early Stage of Paramyxovirus Fusion Activation during Viral Entry. PLoS Pathog..

[23] Welsch JC (2013). Fatal Measles Virus Infection Prevented by Brain-Penetrant Fusion Inhibitors. J. Virol..

[24] Higgins CD, Koellhoffer JF, Chandran K, Lai JR (2013). C-peptide inhibitors of Ebola virus glycoprotein-mediated cell entry: Effects of conjugation to cholesterol and side chain–side chain crosslinking. Bioorg. Med. Chem. Lett..

[25] Li C-G (2013). A Cholesterol Tag at the N Terminus of the Relatively Broad-Spectrum Fusion Inhibitory Peptide Targets an Earlier Stage of Fusion Glycoprotein Activation and Increases the Peptide’s Antiviral Potency *In Vivo*. J. Virol..

[26] Pessi A (2015). Cholesterol-conjugated peptide antivirals: a path to a rapid response to emerging viral diseases. J. Pept. Sci..

[27] Pardridge WM (2012). Drug transport across the blood-brain barrier. J. Cereb. Blood Flow. Metab..

[28] Madsen K (2007). Structure-activity and protraction relationship of long-acting glucagon-like peptide-1 derivatives: importance of fatty acid length, polarity, and bulkiness. J. Med. Chem..

[29] Pocai A (2009). Glucagon-like peptide 1/glucagon receptor dual agonism reverses obesity in mice. Diabetes.

[30] Santoprete A (2011). DPP-IV-resistant, long-acting oxyntomodulin derivatives. J. Pept. Sci..

