# Supplementary material for: A first-in-human study of the novel HIV-fusion inhibitor C34-PEG4-Chol
Source: Sci Rep. 2017 Aug 25;7:9447. doi: 10.1038/s41598-017-09230-0 (PMC5572697; doi:10.1038/s41598-017-09230-0)
Supplement: Supplementary file 1 — supplementary information [file 41598_2017_9230_MOESM1_ESM.pdf]

**Title:**

A first-in-human study of the novel HIV-fusion inhibitor C34-PEG$_4$-Chol.

**Authors:**

Killian Quinn[1], Cinzia Traboni[2], Sujan Dily Penchala[3], Georgios Bouliotis[4], Nicki Doyle[1], Vincenzo Libri[5], Saye Khoo[3], Deborah Ashby[4], Jonathan Weber[1], Alfredo Nicosia[2,6,] Riccardo Cortese[2,6], Antonello Pessi[2,6,7] * and Alan Winston[1] *.

**Affiliations:**

1. Department of Medicine, Imperial College London, London, UK, W2 1NY
2. JV Bio, Via Gaetano Salvatore 486, 80145 Napoli, Italy
3. Department of Pharmacology, University of Liverpool, Liverpool, UK, L69 3BX
4. School of Public Health, Imperial College London, London, UK
5. Institute of Neurology, University College London, London WC1N 3BG
6. CEINGE, Via Gaetano Salvatore 486, 80145 Napoli, Italy
7. PeptiPharma, Viale Città D'Europa 679, 00144 Roma, Italy

*Joint last author

"This manuscript is dedicated to our colleague and friend Riccardo Cortese, who passed away on April 27, 2017"

**Supplementary Information 1:**

**Table S1: List of injection site reactions (ISR) grading**

| **Symptom** | **Mild (Grade 1)** | **Moderate (Grade 2)** | **Severe (Grade 3)** | **Potentially Life Threatening (Grade 4)** | **Rationale for grading** | **Frequency of mild/moderate ISRs with enfuvirtide in pivotal Phase 3 studies†** |
|---|---|---|---|---|---|---|
| Pain | Does not interfere with activity | Repeated use of non-narcotic pain reliever > 24 hours or interferes with activity | Any use of narcotic pain reliever or prevents daily activity | Emergency department visit or hospitalization | Preventive Vaccine Clinical Trials grading scale | 93.1 |
| Tenderness | Mild discomfort to touch | Discomfort with movement | Significant discomfort at rest | Emergency department visit or hospitalization | Preventive Vaccine Clinical Trials grading scale | 89.8 |
| Erythema / Redness * | 0.5-2.5 cm lasting >24 hours after dosing | 2.6 - 10 cm at any time post dosing during follow up | > 10 cm at any time post dosing during follow up | Necrosis or exfoliative dermatitis at any time post dosing during follow up | Adapted from Preventive Vaccine Clinical Trials grading scale | 90.8 |
| Induration / Swelling ** | 0.5-2.5 cm lasting >24 hours after dosing AND does not interfere with activity | 2.6 - 10 cm at any time post dosing during follow up OR interferes with activity | > 10 cm at any time post dosing during follow up OR prevents daily activity | Necrosis at any time post dosing during follow up | Adapted from Preventive Vaccine Clinical Trials grading scale | 94.4 |
| Nodules/cysts | 0.5-1.0 cm | 1.1-1.5 cm | 1.6-2.0 cm | NA | In the absence of internationally agreed grading system trial specific grading system has been devised | 81.6 |
| Pruritis associated with injection | Itching localized to injection site AND relieved | Itching beyond the injection site but not generalized OR Itching localized to injection | Generalized itching causing inability to perform usual social & functional activities | NA | DAIDS grading table | Not reported |

| | | |
|---|---|---|
| | spontaneously or with < 48 hours treatment | site requiring ≥ 48 hours treatment |

* In addition to grading the measured local reaction at the greatest single diameter, the measurement should be recorded as a continuous variable.

** Induration/Swelling should be evaluated and graded using the functional scale as well as the actual measurement.

† Percentages are based on T-20 vs. Optimised Regimen Only Studies (TORO-1 and TORO-2), Phase III studies comparing the efficacy and safety of 24 weeks of treatment with enfuvirtide in combination with an optimised background antiretroviral regimen with the efficacy and safety of optimised background therapy alone.

**Supplementary Information 2:**

**Preclinical Pharmacokinetics of C34-PEG$_4$-Chol**

The preclinical PK of C34-chol in mice has already been reported[1]. These results have been repeated and extended to other species for C34-PEG$_4$-Chol. C34-PEG$_4$-Chol was formulated in 10 mM phosphate buffered saline (pH 7.2). The concentration of C34-PEG$_4$-Chol in plasma samples was determined by LC-MS as previously described[1].

**Reference:**

1. Ingallinella P, *et al.* Addition of a cholesterol group to an HIV-1 Peptide Fusion Inhibitor dramatically increases its antiviral potency. *Proc Natl Acad Sci USA* **106**, 5801-5806 (2009).

**Mouse PK**.

The PK of C34-PEG$_4$-Chol in C57BL/6 mice following intravenous (IV) and subcutaneous (SC) dosing is shown in Figure S1, and the calculated PK parameters are reported in Table S1.

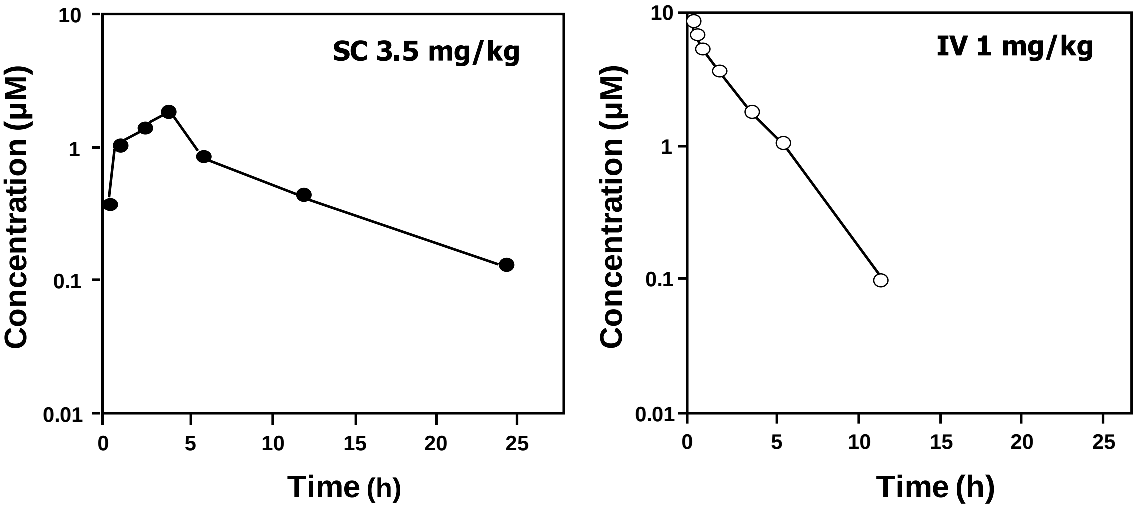


**Figure S2:** ***Pharmacokinetics of C34-PEG$_4$-Chol in mice***. *3.5 mg/kg or 1.0 mg/kg (SC and IV, respectively) of C34-PEG$_4$-Chol in 10mM phosphate buffered saline (PBS) were administered to C57BL/6 mice: volume of administration 5 mL/kg and 2 mL/kg for SC and IV administration, respectively. The concentration of the dosing solution was determined immediately after dosing with the same analytical method utilized to monitor peptide plasma concentration with time, and the analytical curves were adjusted accordingly (SC dose 0.56 mg/mL, theor. 0.5 mg/mL; IV dose 0.62 mg/mL, theor. 0.7 mg/mL). Each data point is the mean of three animals.*

**Table S2**. *Calculated PK parameters of C34-PEG$_4$-Chol in mice, following intravenous (IV) and subcutaneous (SC) administration*.

| Dose (IV); *(mg/kg)* | $AUC_{0\to\infty}$; *(μM·h)* | Cl; *(mL/min/kg)* | Vdss; *(L/kg)* | $t_{½}$; *(h)* |
|---|---|---|---|---|
| 1.0 | 10.78 | 0.29 | 0.05 | 1.97 |
| Dose (SC); *(mg/kg)* | $AUC_{0\to\infty}$; *(μM·h)* | $C_{max}$; *(μM)* | $T_{max}$; *(h)* | F; *(%)* |
| 3.5 | 11.53 | 1.12 | 5.3 | 30.3 |

**Rat PK**.

The PK of C34-PEG$_4$-Chol in Wistar Han rats following IV and SC dosing is shown in Figure S2, and the calculated PK parameters are reported in Table S2.

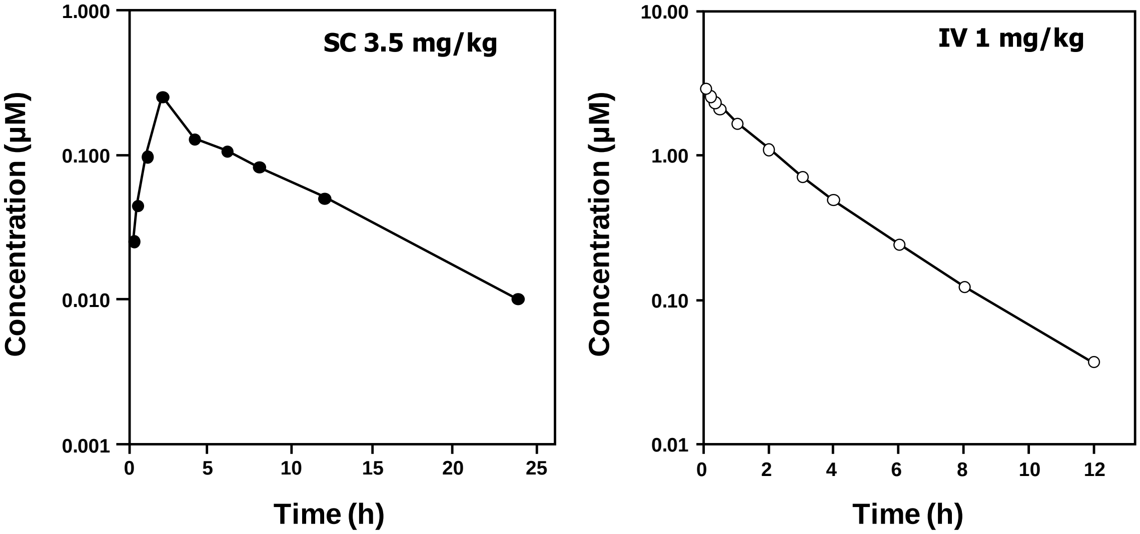


**Figure S3**. ***Pharmacokinetics of C34-PEG$_4$-Chol in rats*** *3.5 mg/kg or 1.0 mg/kg (SC and IV, respectively) of C34-PEG$_4$-Chol in 10mM phosphate buffered saline (PBS) were administered to Wistar Han rats: volume of administration 0.5 mL/kg. The concentration of the dosing solution was determined immediately after dosing with the same analytical method utilized to monitor peptide plasma concentration with time, and the analytical curves were adjusted accordingly. Each data point is the mean of three animals.*

**Table S3**. *Calculated pharmacokinetics parameters of C34-PEG$_4$-Chol in rats, following intravenous (IV) and subcutaneous (SC) administration*.

| Dose (IV); *(mg/kg)* | $AUC_{0\to\infty}$; *(μM·h)* | Cl; *(mL/min/kg)* | Vdss; *(L/kg)* | $t_{½}$; *(h)* |
|---|---|---|---|---|
| 1.0 | 6.6 | 0.48 | 0.08 | 2.18 |
| Dose (SC); *(mg/kg)* | $AUC_{0\to\infty}$; *(μM·h)* | $C_{max}$; *(μM)* | $T_{max}$; *(h)* | F; *(%)* |
| 3.5 | 1.86 | 0.26 | 3.3 | 7.97 |

**Monkey PK**.

The PK of C34-PEG$_4$-Chol in rhesus monkeys following IV and SC dosing is shown in Figure 3, and the calculated PK parameters are reported in Table S3.

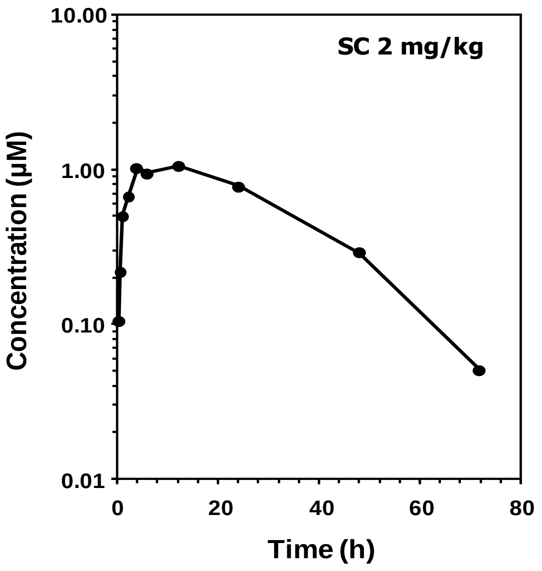


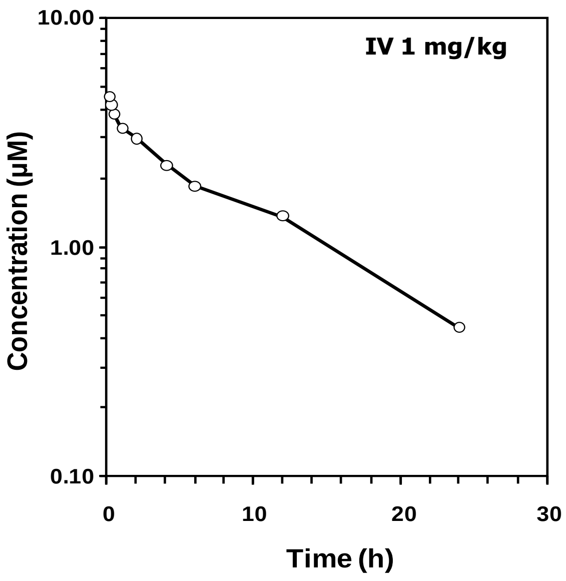


***Figure S4*. *Pharmacokinetics of C34-PEG$_4$-Chol in rhesus monkeys*** *2.0 mg/kg or 1.0 mg/kg (SC and IV administration, respectively) of C34-PEG$_4$-Chol in 10mM phosphate buffered saline (PBS) were administered to rhesus monkeys: volume of administration 0.5 mL/kg. The concentration of the dosing solution was determined immediately after dosing with the same analytical method utilized to monitor peptide plasma concentration with time, and the analytical curves were adjusted accordingly. Each data point is the mean of three animals.*

**Table S4**. *Calculated pharmacokinetics parameters of C34-PEG$_4$-Chol in rhesus monkeys, following intravenous (IV) and subcutaneous (SC) administration.*

| Dose (IV); *(mg/kg)* | $AUC_{0\to\infty}$; *(µM·h)* | Cl; *(mL/min/kg)* | Vdss; *(L/kg)* | $t_{½}$; *(h)* |
|---|---|---|---|---|
| 1.0 | 41.5 | 0.078 | 0.055 | 8.5 |
| Dose (SC); *(mg/kg)* | $AUC_{0\to\infty}$; *(µM·h)* | $C_{max}$; *(µM)* | $T_{max}$; *(h)* | F; *(%)* |
| 2.0 | 37.5 | 1.15 | 6.7 | 45.3 |
